# Early Adverse Family Experiences and Elevated Adrenocorticotropic Hormone Predict Non-Suicidal Self-Injury in Females with Non-Psychotic Mental Disorders and Suicidal Ideation

**DOI:** 10.3390/biomedicines11123181

**Published:** 2023-11-29

**Authors:** Mikhail S. Zinchuk, Tatiana A. Druzhkova, Sofya B. Popova, Marina Y. Zhanina, Alla B. Guekht, Natalia V. Gulyaeva

**Affiliations:** 1 Moscow Research and Clinical Center for Neuropsychiatry, 115419 Moscow, Russia; mzinchuk@mail.ru (M.S.Z.); sopheternity@mail.ru (S.B.P.); m.u.kasatkina@gmail.com (M.Y.Z.);; 2 Department of Functional Biochemistry of Nervous System, Institute of Higher Nervous Activity and Neurophysiology, Russian Academy of Sciences, 117485 Moscow, Russia; 3 Department of Neurology, Neurosurgery and Medical Genetics, Pirogov Russian National Research Medical University, 119049 Moscow, Russia

**Keywords:** non-suicidal self-injury (NSSI), non-psychotic mental disorder, suicidal ideation, stress, hypothalamic–pituitary–adrenal (HPA) axis, cortisol, adrenocorticotropic hormone (ACTH), brain-derived neurotrophic factor (BDNF), early adverse experience

## Abstract

Nonsuicidal self-injurious behavior (NSSI), prevalent in patients with non-psychotic mental disorders (NPMD), is associated with numerous adverse outcomes. Despite active research into the clinical and psychological aspects of NSSI, the underlying biological mechanisms remain obscure. Early adverse experiences are believed to induce long-lasting changes in neuroendocrine mechanisms of stress control playing a key role in NSSI development. The aim of the study was to evaluate parameters potentially predicting development of NSSI in female patients with NPMD and suicidal ideation. Eighty female patients over 18 years with NPMD and suicidal ideation (40 with and 40 without NSSI) and 48 age matching women without evidence of mental illness (healthy controls) were enrolled. Diagnostic interviews and self-report measures were used to assess childhood maltreatment, presence, frequency, and characteristics of suicidal and self-injurious thoughts and behaviors, the Beck Depression Inventory scale to assess severity of depression. Hypothalamic-pituitary-adrenal axis markers, hormones, and neurotrophic factors were measured in blood serum. The likelihood of developing NSSI in patients with NPMD and suicidal ideation was associated with early adverse family history and elevated adrenocorticotropic hormone levels. Dysregulation of hypothalamic-pituitary-adrenal axis as a result of early chronic stress experiences may represent critical biological mechanism promoting the development of NSSI behaviors in patients with NPMD.

## 1. Introduction

Nonsuicidal self-injury (NSSI) is characterized by the intentional destruction of one’s own body tissue without suicidal intent and for purposes not socially sanctioned. NSSI typically begins in adolescence and the most common motives for self-harm are to reduce negative emotions, resolve interpersonal difficulties or induce positive feelings [1]. NSSI is believed to be a complex behavior that emerges through the intersecting effects of social, psychological and biological mechanisms. Recent studies have demonstrated the advantages of conceptualizing NSSI as a transnosological construct. For example, Wang and Eaton [2] found that transdiagnostic dimensions were superior to common DSM-IV and DSM-5 diagnoses in predicting NSSI, accounting for 33.6–38.7% of the variance in NSSI across the sample. These recent findings on NSSI are consistent with data on suicidal behavior, which may also exist across diagnostic boundaries, since suicide-related variables are more strongly associated with the shared variance of multiple mental disorders than with their unique disorder-specific variances [3,4].

Although the purpose of NSSI at the time of harm is, by definition, different from suicide, a history of NSSI has been shown to be an important risk factor for both future suicide attempts and completed suicides [5,6]. These risks increase dramatically when NSSI is co-occurring with mental disorders. Notably, there is a bidirectional relationship between NSSI and mental disorders: the risk of developing non-psychotic mental disorders (NPMD) (personality disorders, bipolar disorder, eating disorders, etc.) is higher among those who have engaged in self-harm, and vice versa [7,8]. Some studies suggest that the comorbidity between depression and NSSI is very high and that depression is one of the most important clinical risk factors for the development of NSSI [9]. Depression and NSSI have been shown to have an interactive effect on suicide risk that is higher than either depression or NSSI alone [10]. Among several possible explanations for these data, it should be noted that patients with NSSI comorbid with depression tend to have poorer interpersonal and family relationships, more negative and stressful life events, a more frequent family history of mental illness, and a more severe clinical course [11,12]. 

Unfortunately, the lack of extensive knowledge about the biological mechanisms underlying NSSI hampers the prevention of suicide remaining one of the leading causes of death among adolescents and young adults worldwide [13,14,15,16,17]. A strong relationship was revealed between a corresponding pattern of the hypothalamic-pituitary-adrenal (HPA) axis dysregulation and chronic stress experiences [18], particularly those experiences that are regarded as an important risk factor for the development of NSSI [19]. Long-lasting activation of HPA axis and the associated release of its executive hormone, cortisol, may be particularly critical during the sensitive period of adolescent development, when glucocorticoids can interfere with the development of brain structures critical for emotional control [20]. Changes in the functioning of the hypothalamic-pituitary-adrenal (HPA) axis may be one of the biological mechanisms contributing to the development and maintenance of NSSI behavior [21,22]. 

Although a large number of previous studies have focused on different aspects of NSSI, the results are often controversial and it is difficult to draw definite conclusions concerning the biological mechanisms of NSSI. This could be explained by the heterogeneity of the study cohorts due to the use of different screening instruments for NSSI and the reliance on the categorical approach to mental disorders as presented in ICD-10 and the main sections of DSM-5. Given the high comorbidity of NSSI with different mental disorders, which in turn are comorbid with each other, we decided to include in our study patients with different NPMDs. In this study, we decided not to include patients with primary psychotic disorders, since previous studies in this population have shown the importance of psychotic disorder-specific factors in the development of NSSI. For example, the study by Güney et al. [23] showed that in a multivariate model, a one-unit increase in the General Psychopathology Subscale score of the Positive and Negative Syndrome Scale (PANSS) was associated with a 1.173-fold increase in odds of NSSI in schizophrenia patients. In contrast, in this study, many variables previously found to be significant in people with NPMD (age, gender, age at onset, forensic history, tattooing) failed to predict NSSI in psychotic patients. Another aspect worth noting is that patients with schizophrenia have many peculiarities in stress markers, hormones and neurotrophic factors that distinguish them from people without psychotic disorders. Taking all this into account, we believe that predictors of NSSI in people with primary psychotic disorders should be studied separately from those with NPMD [24].

Considering data from previous studies showing that most NPMDs, suicide attempts, and NSSI are more common in women than in men [25,26,27], we used data from women only in the current study. We focused on patients with NPMD and suicidal ideation because of the increased risk of suicide in this population, particularly among those who also engage in NSSI. Therefore, identifying factors, including those related to the HPA axis, associated with NSSI in this high-risk population seems particularly important, as this could be a step forward in developing early diagnosis and personalized treatment approaches for this cohort.

The aim of the study was to evaluate parameters that may predict the development of NSSI in female patients with NPMD and suicidal ideation. 

## 2. Materials and Methods

### 2.1. Setting and Subjects

This study was conducted from September 2019 to October 2020 in the Moscow Research and Clinical Center for Neuropsychiatry, the largest clinic in Moscow specializing in the treatment of patients with non-psychotic mental disorders providing free services to permanent residents of Moscow, both via self-referral and via referral from a general practitioner.

All procedures involving human subjects were conducted in accordance with the set of ethical principles presented in the Seventh Revision of the Declaration of the World Medical Association [28]. Thise study was conducted in accordance with local legislation and institutional requirements; the protocol of this study was approved by the Research Ethics Committee of the Moscow Research and Clinical Center for Neuropsychiatry (approval No. 42, 19 August 2019).

The inclusion criteria were as follows: diagnosis of an NPMD according to ICD-10 criteria, suicidal ideation, age 18–35 years, ability to give informed consent and comply with the study protocol. The reason for the upper age limit of 35 years was determined by our aim to focus on patients with current NSSI, which rarely occurs after this age. Our further experience supports this statement, since only one patient (46 years old) was positively screened for current NSSI during the whole enrolment period. The exclusion criteria were as follows: cognitive impairment (score less than or equal to 24 on the Mini-Mental State Examination (MMSE)) [29], current psychotic disorder or lifetime diagnosis of primary psychotic disorder, alcohol or substance use disorders, severe comorbid somatic (e.g., diabetes mellitus, autoimmune or oncologic diseases) or neurologic disorders (e.g., Alzheimer’s and Parkinson’s diseases).

Patients received appropriate medication (treatment as usual) prescribed by a psychiatrist.

### 2.2. Procedure and Instruments

According to the criteria described above, 40 female patients aged 18–35 years with NPMD, suicidal ideation, and NSSI were enrolled (cases—NSSI group). After the enrolment of cases was completed, we also enrolled controls (case-control ratio 1:1)—40 female patients of the same age as the case group participants with NPMD and suicidal ideation but without NSSI (controls—non-NSSI group). 

All patients were seen by an experienced psychiatrist to confirm the diagnosis of NPMD and completed the MMSE. In addition, on the day of admission, all patients were interviewed about NSSI and suicidal behavior using relevant sections of the Russian version of Self-Injurious Thoughts and Behaviors Interview (SITBI) [26,30]. Socio-demographic and clinical data were collected using an ad hoc case report form. On the first three days, all participants completed the Russian versions of the following self-report instruments: the Beck Depression Inventory (BDI) [31,32], the State and Trait Anxiety Inventory (STAI) [33,34], the Brief Reasons for Living Inventory (bRFL) [35,36], and the brief form of the Personality Inventory for DSM-5 (PID-5-BF) [37,38] and the Child Abuse and Trauma Scale (CATS) [39,40].

In addition, 48 generally healthy women of similar age with no current or past history of mental disorders were enrolled as healthy controls (HC) for comparative assessment of average normal levels of biochemical parameters. Female employees of similar age who consented to participate in the study were recruited after a routine medical examination, including a psychiatric evaluation.

Informed consent was obtained from all participants enrolled in the study. Participants provided written informed consent prior to any procedure included in the study protocol.

### 2.3. Samples

Biochemical and hormonal parameters were measured in blood serum obtained from fasting morning venous blood. Samples were collected in Gel/Clotting activator S-Monovette tubes and centrifuged at 2000× *g* for 10 min at 8 °C on an Allegra X-30R Centrifuge (Beckman Coulter, Brea, CA, USA). 

### 2.4. Assessment of Biochemical Indices and Hormones

Cortisol, thyroid-stimulating hormone (TSH), prolactin, and testosterone levels were measured in blood serum through competitive enzyme immunoassay using appropriate kits (Beckman Coulter, Brea, CA, USA) and an ACCESS^®^ 2 immunoassay system (Beckman Coulter, Brea, CA, USA). Adrenocorticotropic hormone (ACTH) was measured using enzyme immunoassay kits from Biomerica (Irvine, CA, USA). Brain-derived neurotrophic factor (BDNF) levels were determined through enzyme-linked immunosorbent assay (ELISA) in blood serum using appropriate Quantikine ELISA test systems (R&D Systems, Minneapolis, MN, USA). BDNF and ACTH levels were measured on an automated enzyme immunoassay analyzer (ChemWell 2910, Awareness Technologies Inc., Palm City, FL, USA). Routine biochemical parameters were determined in blood serum on an automated biochemical analyzer Beckman Coulter AU 680 (Beckman Coulter, Brea, CA, USA) using appropriate kits (Beckman Coulter, Brea, CA, USA). Complete blood count with differential white blood cell count (CBC with diff) and hemogram were performed on an automated analyzer LH-500 (Beckman Coulter, Brea, CA, USA). 

### 2.5. Statistical Analysis

Statistical analyses were performed using STATISTICA 10.0 (StatSoft Inc., Tulsa, OK, USA), GraphPad Prism version 9.4.1 (GraphPad Software, Inc., San Diego, CA, USA), and the R programming environment on the RStudio version 2023.06 platform 2 (2009–2023, Posit Software, PBC, Boston, MA, USA) with the following libraries: ggplot2, ROCR, dplyr, tidyr, MASS, caret, margins. The normality of distributions was assessed using Shapiro–Wilk and Kolmogorov–Smirnov tests. Fisher’s exact test was used to compare qualitative data. To compare quantitative data between multiple unrelated groups, either the ANOVA test with post hoc analysis using Tukey’s test or the Kruskal–Wallis test with post hoc analysis using Dunn’s test was used, depending on the distribution. The Mann–Whitney test was used to compare two independent samples. Data in tables are presented as mean with SD, median with interquartile range, or percentage. Differences were considered significant at *p* < 0.05. Predetermined sample size calculation was performed before the study as well as power analysis of the data obtained. The power analysis for all predictors in one-way ANOVA showed average power ≥ 0.9. The backward logistic regression model was used with the significance level for each variable included set at 0.05. A logistic regression model included several independent (predictor) variables (nominal or continuous) that can be used to predict a dependent (outcome) binominal variable.

## 3. Results

### 3.1. Clinical Characteristics of the Patients

The cohort consisted of patients with the following current diagnoses: personality disorders (34%), depressive disorders (30%), bipolar affective disorders (15%), neurotic and stress-related disorders (13%), mental disorders caused by brain damage and dysfunction (7%). Almost 36% of patients had two or more co-occurring diagnoses, the most common being eating disorders (32%). 

The patients in the two groups compared did not differ in age or socio-demographic characteristics. All patients had suicidal ideation and 38% of patients in each group had attempted suicide in their lifetime. In the NSSI group, almost a quarter of patients had a family history of NSSI compared to 5% in the no history of NSSI group (*p* < 0.05).Both groups were similar in terms of psychiatric diagnoses, with the exception of a significantly lower proportion of patients with neurotic and stress-related disorders in the NSSI group as compared to participants without NSSI experience.Depression (BDI) and trait anxiety (STAI-t) scores were significantly higher in patients with NSSI (Table 1).

### 3.2. Biochemical Characteristics of the Patient and Control Groups

Hormone levels (cortisol, ACTH, prolactin, testosterone) in patients of the NSSI− and NSSI+ groups were significantly increased, whereas BDNF concentration, neutrophil/lymphocyte ratio, and Hb levels were significantly decreased as compared to the respective indices in healthy subjects (Table 2). As compared to patients without NSSI, those with NSSI had significantly higher ACTH levels and significantly lower BDNF levels (Table 2).

### 3.3. Distribution of Clinical and Biochemical Parameters According to the Importance of Their Influence on the Development of NSSI in Patients with NPMD

**Figure 1:**
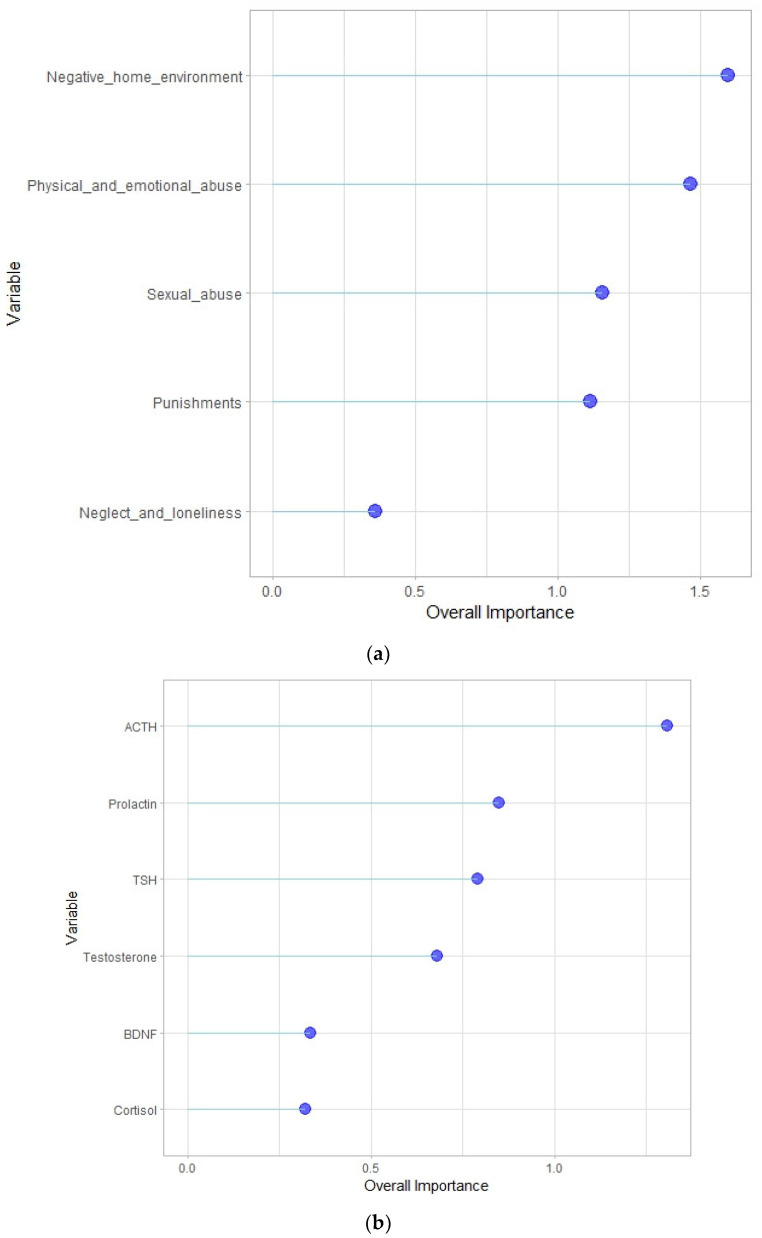
Levels of importance for factors influencing NSSI according to the CATS scale (**a**) and biochemical indices (**b**). An overall importance scale in arbitrary units was used.

The regression model assessing the factors influencing the development of NSSI was constructed using the Russian version of the CATS subscales such as ‘physical and emotional abuse’, ‘sexual abuse’, ‘neglect and loneliness’, ‘negative home environment’ and ‘punishment’ as variables. The varImp function in the R programming environment was used to assess the importance of the variables in the regression model. The real meaning of such a function is the importance of a variable, in particular using an indicator that quantifies the contribution of one or a set of input variables, for the uncertainty of the model [41]. Factor levels were grouped into one overall importance value. This definition was appropriate for our method of examining the importance of variables. The graph (Figure 1a)showed that the factors ‘negative home environment’ and ‘physical and emotional abuse’ had the greatest impact on the accuracy of the regression model. Building a similar model using biochemical parameters, in particular ACTH, prolactin, TSH, testosterone, cortisol and BDNF selected as variables, demonstrated that levels of anterior pituitary hormones (ACTH, prolactin, TSH) were most important factors influencing NSSI (Figure 1b).

### 3.4. Model for Predicting the Probability of Developing NSSI in Patients with NPMD

The model was constructed using logistic regression. The BDI and STAI scale scores, each of the CATS subscale scores (physical and emotional abuse, sexual abuse, neglect and loneliness, negative home environment, punishment), hormone levels (ACTH, pg/mL; cortisol, nmol/L; TSH, Ulu/mL; prolactin, ng/mL; testosterone, nmol/L), and BDNF concentration (ng/mL) concentration were used as predictors (independent variables) to build the model.

**Figure 2:**
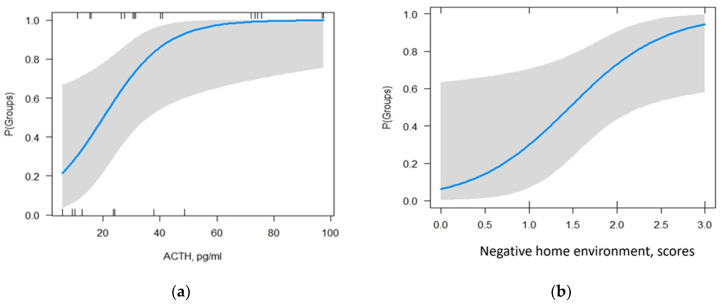
Dependency of the probability of developing NSSI on ACTH level (**a**) and on CATS “negative home environment” subscale score (**b**). The gray area along the blue line indicates a 95% confidence interval.

The coefficient values in the summary table of the model show that only the variables ACTH level and CATS subscale ‘negative home environment’ had a statistically significant effect on the occurrence of NSSI development in patients with NPMD and suicidal ideation. The McFadden pseudo-R-square used for this model was 0.55. This indicates that the model fits the data well and has a good predictive power. The dependence of the probability of developing NSSI on the levels of the predictors is shown in Figure 2 (ACTH level, Figure 2a; CATS “negative home environment” subscale score, Figure 2b).

According to this model, an increase in ACTH of 10 pg/mL augments the odds of NSSI in patients by a factor of 11, and an increase in the CATS “negative home environment” subscale by 1 unit raises the odds of NSSI in patients by a factor of 6.4 (Table 3).

In accordance with the classification table, using tested data from 25 patients (31% of the total cohort), the sensitivity of the model was 94% and the specificity was 100%. The accuracy of the selected model with a cut-off of 0.5 for binary classification was 96%. The area under the curve was 0.9. These data indicate a good performance of the model selected.

## 4. Discussion

The aim of the current study was to assess HPA axis parameters associated with NSSI in female patients with NPMD. The cohort consisted of 34% with personality disorders, 30% with depressive disorders, 15% with bipolar disorder, 13% with other neurotic and stress-related disorders and 7% with mental disorders caused by brain damage and dysfunction. Patients with NSSI had significantly higher levels of trait anxiety and higher scores on the Beck Depression Scale. In this group of patients, the percentage of women with a family history of NSSI was five times higher than in patients without NSSI, which may indicate a predisposition to NSSI [42,43].

The biochemical results obtained suggest that the levels of stress hormones (ACTH, cortisol) were significantly elevated in patients of both NPMD groups, while the concentration of BDNF and the neutrophil/lymphocyte ratio were significantly reduced as compared to the corresponding parameters of healthy individuals. These data are consistent with most of the available literature and generally confirm that the HPA axis, the neurotrophic factor system, and cell-mediated inflammation are involved in the pathophysiology of patients with NPMD. The HPA axis is a major neuroendocrine system that controls responses to stress and several other major bodily processes, including digestion, immunity, mood and emotion, sexuality, and energy storage and expenditure. It is well known that HPA axis with cortisol as its main output, orchestrate the development of psychopathologies [44]. Previous studies examining associations between NSSI and the HPA axis in patients with major depressive disorder (MDD) have found an altered pattern of HPA axis regulation [45,46]. Most importantly, the data showed that depressed individuals with NSSI had the lowest levels of salivary cortisol in response to the Trier Social Stress Test accompanied by the highest ratings of observed stress as compared to patients with depression and healthy persons [22]. In the current study, no significant difference in cortisol levels was found between the group of patients with NSSI and those without NSSI, possibly due to increased cortisol production in both groups. Notably, cortisol levels were slightly higher in patients with NSSI, but did not reach the level of significance though showing a statistical trend (*p* = 0.06).

An important result of this study is revealing significantly higher ACTH levels and significantly lower BDNF levels in patients with NSSI as compared to those without NSSI. This may indicate a more pronounced HPA axis dysregulation potentially leading to subsequent damage to sensitive brain structures, including the hippocampus, and more prominent disturbances in neuroplasticity in patients with NSSI. Previously, HPA axis disturbances have been shown to affect memory function in patients with MDD, post-traumatic stress disorder, and borderline personality disorder [47]. In addition, NSSI and mental disorders including, depression, anxiety, and post-traumatic stress disorder, are known to be associated with HPA axis and prefrontal cortex dysfunction, suggesting a functional link between aberrant prefrontal corticosteroid signaling, stress adaptation, emotional control, mood regulation, and pathways mediating stress-related psychopathology [48]. The difference in both ACTH and BDNF levels found in our study between patients with and without NSSI seems to be important and may be the basis for further research on the mechanisms underlying comorbidity of mental disorders and NSSI.

The distribution of biochemical parameters of the patients according to their importance for NSSI suggests that the anterior pituitary hormones (ACTH, prolactin, TSH) may be tightly involved in its development (Figure 1b), with ACTH playing the leading role. Previously, it was shown that the level of prolactin, vulnerable to the influence of different stressors, has a regulatory effect on the adrenal glands, acting as a synergist of ACTH, and, accordingly, modifying the stress response [49]. These findings suggest that the development of NSSI in patients with NPMD may be associated with HPA axis dysregulation at the pituitary level. The pituitary gland is known to play an integral role in mediating stress response through its involvement in HPA axis function. The pituitary gland is under the influence of hypothalamic corticotropin-releasing hormone and secretes ACTH acting in a feedback loop to propagate the stress response; thus, pituitary may be a region critically affected by failure in stress control. Some studies suggest that pituitary gland volume (PGV) is altered in individuals with stress-related psychopathology which may be associated with age-related changes in PGV during adolescence [50,51]. A recent study examining pituitary PGV by magnetic resonance imaging in adolescents with NSSI as compared to age-matched healthy controls provided preliminary evidence of alterations in pituitary maturation in adolescents engaged in NSSI [52].

All patients enrolled in our study had suicidal ideation. It was found that the age of onset of suicidal ideation in the group of patients without NSSI was 18.0 (13.8; 22.0) years, while in patients with NSSI it was 14.0 (13.0; 16.3) years, and the age of onset of NSSI was 14.5 (13.0; 18.0) years. Our results support the view that patients with suicidal ideation and NSSI represent a more severe clinical group than those without NSSI, characterized by an earlier (adolescent) onset of suicidal ideation. The association between NSSI, adolescence and female gender is consistent with the importance of the role played by biological hormonal components, particularly pituitary hormones. Adolescence is a transitional period, defined as the period between the onset of puberty and the beginning of independence, and is characterized by continued structural maturation of the brain and hormonal changes that begin at puberty [53]. This period coincides with the onset of HPA axis reactivity to psychological stress, sex differences and the behavioral expression of negative emotions in response to stressful situations [54]. Stress-responsive regions of the brain, such as the fronto-cortical and limbic areas, are still maturing during adolescence [55]. Stress responses emerging during this period of life are accompanied by close interactions between stress hormones, the increase in HPA axis responsiveness and the gonadal hormonal axes [56]. Brain structures mediating emotions and cognitive functions, particularly the hippocampus, are known to be most vulnerable to changes in HPA axis activity associated with early stress experiences [57,58].

In the current study, we examined the effects of a number of different types of early life stressors (i.e., childhood and adolescence), including physical and emotional abuse, sexual abuse, neglect and loneliness, negative home environment, and punishment, on the development of NSSI in female patients with NPMD. It was found that the factors ‘negative home environment’ and ‘physical and emotional abuse’ had the greater impact on the development of NSSI (Figure 1a). The most important factor increasing the likelihood of developing NSSI in such patients was an adverse family environment, according to data from the CATS subscale ‘negative home environment’ (Figure 2b). This subscale includes statements about family relationships: abuse, violence, feelings of unhappiness, alcoholism/drug addiction in the family, feelings of security in the family, the ability to freely invite someone to home, and a question about the general feeling of having had a difficult childhood. It can be assumed that people whose mental disorder was exacerbated by NSSI had previously experienced prolonged exposure to stress. Simultaneously, most of them had a family history of NSSI. A number of studies suggest that during sensitive periods in genetically predisposed individuals, prolonged exposure to stress may have a greater impact on the developmental trajectory of brain structures as compared with potentially stronger but shorter-term stressors [20].

The results obtained in the current study using the backward logistic regression method indicate that the likelihood of developing NSSI in patients with NPMD is independently associated with early adverse family experiences and elevated ACTH levels (Figure 2, Table 3). More than half of the studies mentioned that child maltreatment, especially emotional abuse and emotional neglect, may contribute to the development of NSSI in patients with mental disorders [59,60,61,62]. It has been suggested that exposure to psycho-emotional trauma in childhood and adolescence may trigger psychiatric disorders by altering the function of the HPA axis [63,64]. Previous research has also found links between early life stress and changes in PGV growth during adolescence [65,66].

Developmental plasticity is usually maximal during immature stages of development, and the neuroendocrine stress system might be expected to be most vulnerable to chronic stress [67]. The negative effects of chronic stress are likely to be mostly due to its prolonged duration, overriding the highly dynamic regulation critical for optimal HPA axis function. Some studies have also shown that chronic stress is associated with hyperactivation of HPA axis [68]. Glucocorticoids, executive hormones of HPA axis ensure the coordinated functioning of key components and mechanisms of brain plasticity at different levels, including neurogenesis, glutamatergic neurotransmission, microglia and astrocytes, systems of neurotrophic factors, neuroinflammation, etc. [69]. Adaptation to chronic hyperactivation of the HPA axis may lead to loss of the system sensitivity through chronic activation of negative feedback and feed-forward regulators. The results of some studies have shown a reciprocal relationship between chronic emotional stress and engagement in NSSI [70]. It has been suggested that chronic stress in adolescence promotes the development of atypical regulation of the HPA axis, leading to increased emotional vulnerability in adulthood.

## 5. Strengths and Limitations of this Study

Our study has both strengths and limitations.

The first strength is that the predictors of NSSI were studied in a high-risk population (people with NPMD and suicidal ideation). Participants in both groups (with and without NSSI) had current suicidal ideation and didn’t differ significantly in lifetime suicide attempts. To our knowledge, this is the first study of hypothalamic-pituitary-adrenal axis markers, hormones and neurotrophic factors in a sample of people with NPMD and suicidal ideation with and without NSSI. This may be an important contribution to our knowledge of the problem, since previous studies have shown early stress exposure and HPA dysfunction in people with suicidal ideation and attempts (in our study, these parameters did not differ in the groups studied). We believe that the design we used allowed us to show that NSSI in NPMD is associated with hypothalamic-pituitary-adrenal axis abnormalities, independently of the presence of suicidal ideation and the tendency to progress from suicidal ideation to suicide attempts. 

Another strength of our study is that it addresses the knowledge gap about the role of early stress exposure in the development of NSSI in Russian people, a group that differs in many socio-cultural aspects from both European and Asian populations. 

The main limitations of this study are the rather small cohort of patients enrolled, the inclusion of only non-psychotic patients, the Caucasian sample and the lack of comparison with male patients. Further research is therefore needed in these populations before the findings can be generalized to the whole population of patients with mental disorders and suicidal ideation.

## 6. Conclusions

Our study showed that the likelihood of developing NSSI in female patients with NPMD and suicidal ideationwas increased by elevated levels of ACTH, one of the main players in HPA axis activity, and by early adverse family environment, which may indirectly indicate the presence of chronic stress during immature stages of development. The findings hint that the chronic stress during early life in some people with NPMD may alter the neuroarchitecture in brain regions that regulate the HPA axis, affecting the trajectory of brain development and exacerbating patterns of emotional and cognitive behavior. The results of our study support the importance of routine screening for early adverse experiences in the Russian population, since these individuals are at higher risk of engaging in NSSI behaviors. The latter is closely related to suicidality because it reduces the fear of attempting suicide (mostly through the habitual experience of feeling pain and seeing blood in the process of repeated self-harm). Besides, in advanced clinical settings, testing for early adverse family history and elevated ACTH levels could be used to identify people with NPMD and suicidal ideation who are at high risk of NSSI.

## Figures and Tables

**Table 1:** Sociodemographic and clinical characteristics of patients with and without NSSI.

Parameter/Group	NSSI− (*n* = 40)	NSSI+ (*n* = 40)
Socio-demographic data		
Age (years)	27.5 (22.0; 32.0)	27.5 (22.0; 31.8)
Education (secondary/incomplete higher; higher), %	35/65	28/72
Employed (−/+), %	52/48	50/50
Marital status (single/coupled), %	55/45	50/50
Parent’s family structure (incomplete/complete), %	50/50	45/55
Current diagnosis		
Bipolar affective disorders (F31), %	12	18
Depressive disorders (F32/33), %	30	30
Neurotic and stress-related disorders (F40/41/43/44/45), %	20	5 *
Personality disorders(F60/61), %	31	37
Eating disorders, %	23	40
Mental disorders caused by brain damage and dysfunction (F06/07), %	5	8
Diagnosed with 2 or more mental disorders, %	28	45
Clinical characteristics		
Family history of mental health disorders, %	65	68
Family history of suicidality, %	20	38
Family history of NSSI, %	5	23 *
History of physical abuse, %	65	83
History of bullying at school, %	65	80
Lifetime suicide attempts, %	38	38
Current smoking (nicotine products), %.	56	55
Age at first contact with mental health services, years	22.5 (18.5; 30)	22.0 (18.0; 28.0)
Age of onset of suicidal ideation, years old	18.0 (13.8; 22.0)	14. 0 (13.0; 16.3)
Age of onset of NSSI, years old	-	14.5 (13.0; 18.0)
Psychological scales data		
BDI, scores	27.8 ± 9.4	31.1 ± 9.1 *
STAI (State Anxiety), scores	60.2 ± 9.9	63.8 ± 9.6
STAI (Trait anxiety), scores	59.0 ± 8.4	62.8 ± 8.6 *
bRFL (total), scores	34.4 ± 11.7	39.7 ± 15.3
PID-5-BF (total), scores	34,8 ± 9.2	32.4 ± 11.4
CATS (total), scores	7.4 ± 3.2	9.0 ± 3.4

Data are presented as mean with SD, median with interquartile range, and percentages. Fisher’s exact test was used to compare qualitative data. To compare quantitative data between unrelated groups, either the unpaired *t*-test or Mann–Whitney test was used, depending on the distribution. * *p* < 0.05.

**Table 2:** Laboratory data of patients and control group.

Parameter/Group	HC *n* = 48	NSSI− *n* = 40	NSSI+ *n* = 40
Age	25.0 (23.0; 27.0)	27.5 (22.0; 32.0)	27.5 (22.0; 31.8)
TSH3, ulU/mL	1.7 (1.2; 2.3)	1.7 (1.3; 2.1)	1.9 (1.5; 2.7)
ACTH, pg/mL	15.8 (9.7; 22.2)	24.3 (16.0; 57.0) *^,^**	37.4 (26.7; 56.8) *
Testosterone, nmol/L	1.4 (1.0; 2.1)	2.5 (1.6; 2.9) *	2.1 (1.5; 2.7) *
Prolactin, ng/mL	11.9 (8.2; 15.9)	20.9 (14.8; 34.3) *	19.7 (13.1; 36.1) *
Cortisol, nmol/L	265.9 (192.5; 378.8)	447.6 (381.5; 558.7) *	533.7 (427.1; 600.9) *
BDNF, ng/mL	25.5 ± 5.5	22.1 ± 6.0 *^,^**	18.8 ± 5.7 *
While blood cells, WBCs, 10^3^ µL	6.3 ± 1.6	6.5 ± 1.5	6.0 ± 1.5
Neutrophils, NEI, %/NEI, 10^3^ µL	56.8 ± 10.8/3.6 ± 1.3	50.4 ± 11.3/3.3 ± 1.2 *	49.6 ± 10.0/3.0 ± 1.2 *
Lymphocytes, LY, %/LY, 10^3^ µL	32.2 ± 9.6/2.0 ± 0.7	37.2 ± 10.0/2.4 ± 0.8 *	38.9 ± 9.0/2.3 ± 0.7 *
Monocytes, MO, %	7.7 ± 2.4	8.0 ± 2.2	8.3 ± 2.2
Eosinophils, EO, %	2.4 ± 1.1	3.5 ± 1.9 *^,^**	2.4 ± 1.3
Basophils, BA, %	0.8 ± 0.4	0.9 ± 0.4 **	0.7 ± 0.3
Erythrocytes, RBC, 10^6^ µL	4.9 ± 0.4	4.4 ± 0.3	4.4 ± 0.4
Hemoglobin, Hb, g/L	146.2 ± 13.0	134.2 ± 8.0 *	133.1 ± 9.6 *
Platelets, PLT, 10^3^ µL	248.6 ± 60.3	230.3 ± 52.7	253.1 ± 67.5

Data are presented as mean with SD and median with interquartile range. To compare quantitative data between three unrelated groups, depending on the distribution, either the ANOVA test with post hoc analysis using Tukey’s test or the Kruskal–Wallis test with post hoc analysis using Dunn’s test was used. *p* < 0.05: * compared to HC; ** compared to NSSI+.

**Table 3:** Odds ratio of predictors for model-assessed probability of developing NSSI.

Parameter	Estimate	Std Error	Statistic	*p*-Value
ACTH, pg/mL	1.10	0.04	221	0.027
CATS “negative home environment” subscale, scores	6.37	0.9	2.06	0.039

## Data Availability

The data generated in the present study are available upon reasonable request.

## Abbreviations

ACTH, adrenocorticotropic hormone; BDI, the Beck Depression Inventory; BDNF, brain-derived neurotrophic factor; bRFL, Brief Reasons for Living Inventory; CATS, the Child Abuse and Trauma Scale; DSM, the Diagnostic and Statistical Manual of Mental Disorders; HCs, healthy controls; HPA axis, hypothalamic–pituitary–adrenal axis; ICD-10, International Classification of Diseases 10th Revision; NPMDs, non-psychotic mental disorders; NSSI, non-suicidal self-injury; PID-5-BF, the brief form of the Personality Inventory for DSM-5; PGV, pituitary gland volume; SITBI, Self-Injurious Thoughts and Behaviors Interview; STAI, the State and Trait Anxiety Inventory; TSH, thyroid-stimulating hormone.
